# The World Society of Emergency Surgery (WSES) spleen trauma classification: a useful tool in the management of splenic trauma

**DOI:** 10.1186/s13017-019-0246-1

**Published:** 2019-06-17

**Authors:** Federico Coccolini, Paola Fugazzola, Lucia Morganti, Marco Ceresoli, Stefano Magnone, Giulia Montori, Matteo Tomasoni, Stefano Maccatrozzo, Niccolò Allievi, Savino Occhionorelli, Yoram Kluger, Massimo Sartelli, Gian Luca Baiocchi, Luca Ansaloni, Fausto Catena

**Affiliations:** 1 General, Emergency and Trauma Surgery Department, Bufalini Hospital, Viale Ghirotti 268, 47521 Cesena, Italy; 2 General, Emergency and Trauma Surgery Department, Papa Giovanni XXIII Hospital, Bergamo, Italy; 3 General and Emergency Surgery Department, Sant’Anna University Hospital, Ferrara, Italy; 4 Emergency and Trauma Surgery, Rambam Medical Centra, Haifa, Israel; 5 General Surgery, Macerata Hospital, Macerata, Italy; 6 Department of Clinical and Experimental Sciences, University of Brescia, Brescia, Italy; 7 Emergency Surgery Department, Parma University Hospital, Parma, Italy

**Keywords:** Spleen trauma, Classification, Validation, Practice, Surgery, Outcome, Non-operative management, Quality

## Abstract

**Background:**

The World Society of Emergency Surgery (WSES) spleen trauma classification meets the need of an evolution of the current anatomical spleen injury scale considering both the anatomical lesions and their physiologic effect. The aim of the present study is to evaluate the efficacy and trustfulness of the WSES classification as a tool in the decision-making process during spleen trauma management.

**Methods:**

Multicenter prospective observational study on adult patients with blunt splenic trauma managed between 2014 and 2016 in two Italian trauma centers (ASST Papa Giovanni XXIII in Bergamo and Sant’Anna University Hospital in Ferrara). Risk factors for operative management at the arrival of the patient and as a definitive treatment were analyzed. Moreover, the association between the different WSES grades of injury and the definitive management was analyzed.

**Results:**

One hundred twenty-four patients were included. At multivariate analysis, a WSES splenic injury grade IV is a risk factor for the operative management both at the arrival of the patients and as a definitive treatment. WSES splenic injury grade III is a risk factor for angioembolization.

**Conclusions:**

The WSES classification is a good and reliable tool in the decision-making process in splenic trauma management.

## Introduction

**Fig. 1:**
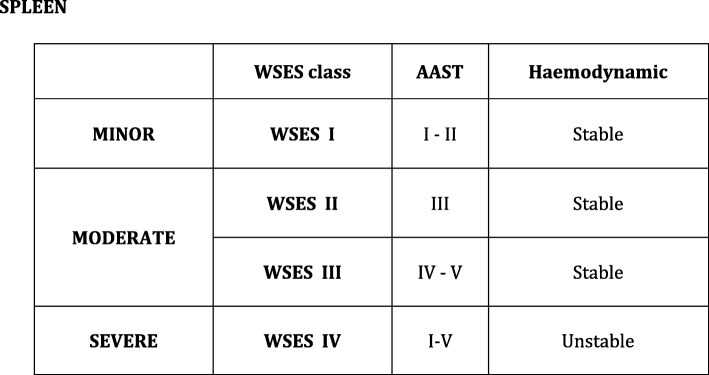
WSES Spleen trauma classification

The most commonly used classification of splenic trauma is the American Association for the Surgery of Trauma (AAST)-Organ Injury Severity Score (OIS). It was initially ideated to allow the comparison between different series of patients; then, it has been used as a classification system to drive treatment strategies. It is based on spleen lesion anatomy [1]. This scale was validated by several studies with large sample sizes [2–4] showing as both the management at the patient arrival (operative management (OM) vs non-operative management (NOM)), and the NOM failure rate was associated with the ASST lesion grade in patients with blunt splenic trauma. In fact, the anatomy of the lesions plays a fundamental role in determining the conditions of the patients. In some situations, however, patient conditions lead to an emergent transfer to the operating room (OR) without the opportunity to define the grade of splenic lesions before the surgical exploration. In these cases, the physiopathologic status of the patients leads the therapeutic decision, more than the anatomy of the splenic lesions. Moreover, there are patients with high-grade splenic lesions without hemodynamic repercussions that can be managed with NOM thanks to the modern tools in bleeding management. As a counterpart, there exists a cohort of patients with hemodynamic instability requiring urgent surgical intervention due to low-grade splenic injuries. In May 2017, during the World Society of Emergency Surgery (WSES) World Congress in Campinas, Brazil, the final version of the WSES guidelines on spleen trauma was approved (Fig. 1) [5]. The WSES grading system takes into account both the patient’s condition and the anatomy of lesions.

The aim of the present study is to evaluate the efficacy and trustfulness of the WSES classification as a tool in decision-making process during spleen trauma management.

## Methods

This is an analysis of two prospectively enrolled adult patient cohorts with blunt splenic trauma managed between 2014 and 2016 in two Italian trauma centers (TC) (ASST Papa Giovanni XXIII in Bergamo and Sant’Anna University Hospital in Ferrara) stratified according to the WSES classification. Ethical committee and patients’ consent to participate were waived because no personal or sensible data were recorded and no specific intervention was adopted other than the usual clinical practice. Patients’ characteristics were collected (age, sex, comorbidity, ASA (American Society of Anesthesiologists) score, antiplatelet or anticoagulant therapy). Trauma mechanism of injury, patient conditions at the arrival in the emergency department (ED) (systolic blood pressure (SBP), heart rate (HR), shock index (SI), need of red blood cell (RBC) transfusion), blood gas test (pH, base excess (BE), lactates (Lac)), blood exams (CBC, platelet count, INR, fibrinogen), and eco-fast results were reported. We defined a patient “hemodynamically unstable” if, after resuscitation in the ED and without vasoactive drugs, he/she had a SBP lower than 90 mmHg, a shock index higher than 1, or a BE lower than − 5.

For patients who underwent CT at the arrival, the AAST classification for the splenic injury, the number of abdominal quadrants with hemoperitoneum, and the presence of vascular lesions (contrast blush (CB), pseudoaneurysm (PSA), arterovenous fistula (AVF)) were reported. For patients who underwent urgent surgical intervention, intraoperative (for splenectomized patients) or postoperative CT findings were registered. The Injury Severity Score (ISS) and the presence of associated abdominal, pelvic, or cerebral lesions were reported. Patients were classified according to the 2017 WSES classification. The management at the arrival (observation, distal angioembolization (AE), proximal AE, splenectomy, intraperitoneal packing, hemostasis of the splenic injury, surgical intervention for other organ lesions), the time between the arrival in the ED and the first urgent intervention, and the need of further intervention during hospital stay (AE or splenectomy) have been recorded.

It was defined OM if the patient underwent urgent surgical intervention at the arrival at the ED and if during the surgical procedure, a splenectomy or a hemostatic splenic technique (e.g., splenic packing or splenorrhaphy) was performed. The NOM could include AE or not. Failure of NOM (fNOM) was defined as the need of performing a splenectomy after starting NOM. To validate the 2017 WSES classification, the risk factors for OM at the arrival of the patient and for OM as a definitive treatment (including both patients treated with OM at the arrival and patients operated for fNOM) have been analyzed. It was verified if the WSES grade was a risk factor for OM at the arrival and as a definitive treatment for adult patients with blunt splenic trauma.

### Statistical analysis

Continuous variables were expressed as mean and standard deviation; categorical data were expressed as proportions and percentages. *t* test was used for continuous variables with normal distribution and the Mann-Whitney test for non-normal distribution variables. Parametric variables were compared with chi-square test. Multivariate models were calculated with the linear logistic regression method including all the variables resulted significantly associated (*p* < 0.05) with the selected outcome at univariate analysis. All the statistical analysis was performed with IBM SPSS 20 (IBM Corp. released 2011; IBM SPSS Statistics for Windows, Version 20.0; Armonk, NY: IBM Corp.).

## Results

The study includes 124 patients older than 17 years with blunt splenic lesion, of whom 66 managed in ASST Papa Giovanni in Bergamo and 58 in Sant’Anna University Hospital in Ferrara. The two groups of patients were similar in terms of epidemiological features, trauma mechanism of injury, ISS, and splenic injury grade. Patient characteristics are reported in Table 1.

**Table 1:** Patient characteristics

Characteristics	*N* = 124 Mean ± SD Median (range)
Age (years)	50.23 ± 18.36
	48.68
	(17.00–91.00)
M/F	91/33
	(73.4%/26.6%)
Trauma mechanism of injury	
-Invested pedestrian	11 (8.9%)
-Car	38 (30.6%)
-Motorbike	39 (31.5%)
-Bike	5 (4.0%)
-Precipitation	17 (13.7%)
-Others	14 (11.3%)
ISS	27.93 ± 13.02
	27.00
	(5.00–75.00)
HR at arrival in ED (bpm)	90.27 ± 20.27
	88.00
	(48.00–145.00)
SBP at arrival in ED (mmHg)	113.91 ± 25.00
	117.00
	(53.00–170.00)
pH	7.31 ± 0.12
	7.33
	(6.80–7.47)
BE (mmol/L)	− 3.23 ± 3.43
	− 2.8
	(−14.50 to + 2.10)
Lac	3.20 ± 1.87
	2.92
	(0.80–9.24)
Hb (g/dL)	12.53 ± 2.53
	12.95
	(3.30–16.80)
INR (s)	1.37 ± 0.72
	1.16
	(0.66–5.93)
Fibrinogen (mg/dL)	231.66 ± 122.74
	210.00
	(26.00–1120.00)
Platelets (× 10^3^/mL)	218.92 ± 72.27
	220.00
	(55.00–460.00)
Number of RBC units transfused in ED	0.48 ± 0.96
	0.00
	(0.00–4.00)
Positive eco-fast	62 (50.0%)
Negative eco-fast negativa	44 (35.5%)
N.A.	18 (14.2%)
AAST 1	3 (3.2%)
AAST 2	48 (38.7%)
AAST 3	34 (27.4%)
AAST 4	30 (24.2%)
AAST 5	5 (4.0%)
N.A.	3 (2.4%)
AAST > 3	35 (28.2%)
AAST ≤ 3	87 (70.2%)
WSES I	44 (35.5%)
WSES II	27 (21.8%)
WSES III	18 (14.5%)
WSES IV	30 (24.2%)
N.A.	5 (4.0%)
WSES IV	30 (24.2%)
WSES < IV	89 (71.8%)
Presence of CB	33 (26.6%)
Absence of CB	74 (59.7%)
N.A.	17 (13.7%)
Presence of PSA/FAV	4 (3.2%)
Absence of PSA/FAV	101 (81.5%)
N.A.	19 (15.3%)
Number of quadrants with hemoperitoneum	1.59 ± 1.45
	1.00 (0.00–5.00)
Associated abdominal and pelvic lesions	58 (46.8%)
No associated abdominal and pelvic lesions	66 (53.2%)
Associated brain injuries	24 (19.4%)
No associated brain injuries	100 (80.6%)

*M/F* male/female, *ISS* Injury Severity Score, *HR* heart rate, *N.A.* not available, *SBP* systolic blood pressure, *ED* emergency department, *BE* base excess, *Lac* lactates, *Hb* hemoglobin, *RBC* red blood cell, *AAST* American Association for the Surgery of Trauma, *WSES* World Society of Emergency Surgery, *CB* contrast blush, *INR* International Normalized Ratio

NOM rate was 53.2% (66 patients) and OM rate 46.0% (58 patients). Among OM patients, we had 84.5% (49 patients) of patients treated with splenectomy and 15.5% (9 patients) with hepatic and splenic packing (in patients with hepatic lesion associated) and/or splenic hemostasis (Table 2).

**Table 2:** Patient outcomes

Variable	*N* = 124 Mean ± SD Median (range)
NOM	66 (53.2%)
OM	58 (46.0%)
-Splenectomy	49 (84.5%)
-Packing/hemostasis	9 (15.5%)
Splenic preservation rate	67 (54.0%)
AE	22 (17.8%)
-Proximal	8 (36.4%)
-Distal	11 (50.0%)
-Distal + proximal	2 (9.1%)
-N.A.	1 (4.5%)
Time between arrival at the ED and the first therapeutic procedure (min)	207.65 ± 295.76
	145.00
	(15.00–1920.00)
Length of ICU stay (days)	9.76 ± 14.94
	5.00
	(0.00–87.00)
Total length of stay (days)	20.01 ± 18.21
	14.00
	(0.50–90.00)
fNOM (*N* = 63)	8 (12.7%)
Complications	47 (41.2%)
Global mortality	13 (10.5%)
Specific mortality	0 (0.0%)

*NOM* non-operative management, *OM* operative management, *N.A.* not available, *fNOM* failure of non-operative management, *ICU* intensive care unit

Among NOM patients, 22 underwent AE (17.8% of total patients and 33.3% of NOM patients) at the arrival or during the hospital stay (Table 2).

Risk factors for OM at the arrival of patient in the ED, including the WSES splenic injury grade, were analyzed with univariate (Table 3) and multivariate (Table 4) analysis.

**Table 3:** Univariate analysis of risk factors for OM at the arrival of patient at the ED

Variable	Mean ± SD Median (range)		*p* value
	NOM	OM	
Age < 55 years	42.3%	57.7%	n.s.
Age > 55 years	50.0%	50.0%	
Age (years)	50.54 ± 18.17	49.87 ± 18.73	n.s.
	49.35 (18.00–91.00)	48.00 (17.00–85.60)	
No anticoagulant/antiplatelet drugs	48.8%	51.2%	n.s.
Anticoagulant/antiplatelet drugs	40.0%	60.0%	
HR (mean ± SD)	85.95 ± 18.66	95.24 ± 21.07	0.009
Median (range) (bpm)	80.00 (48.00–133.00)	95.00 (55.00–145.00)	
HR < 120 bpm	58.7%	41.3%	n.s.
HR > 120 bpm	46.8%	53.2%	
SBP(mmHg)	120.40 ± 21.35	106.51 ± 26.92	0.002
	120.00 (70.00–170.00)	105.00 (53.00–167.00)	
SBP > 90 mmHg	60.4%	39.6%	0.001
SBP < 90 mmHg	21.7%	78.3%	
Shock index < 1	60.2%	39.8%	0.002
Shock index > 1	26.9%	73.1%	
AAST 1	100.0%	0.0%	< 0.001
AAST 2	81.3%	18.7%	
AAST 3	44.1%	55.9%	
AAST 4	26.7%	73.3%	
AAST 5	0.0%	100.0%	
AAST ≤ 3	66.7%	33.3%	< 0.001
AAST > 3	22.9%	77.1%	
WSES I	86.4%	13.6%	< 0.001
WSES II	44.4%	55.6%	
WSES III	44.4%	55.6%	
WSES IV	20.0%	80.0%	
WSES I-II-III	63.8%	36.2%	< 0.001
WSES IV	20.0%	80.0%	
ISS	24.38 ± 12.68	32.05 ± 12.27	< 0.001
	22.00 (5.00–75.00)	29.00 (9.00–66.00)	
ISS < 25	72.0%	28.0%	0.001
ISS > 25	40.9%	59.1%	
Lac	3.01 ± 1.90	3.51 ± 1.85	n.s.
	2.66 (0.80–9.24)	3.08 (1.30–8.00)	
BE (mmol/L)	− 3.34 ± 3.82	− 3.06 ± 2.88	n.s.
	− 2.80 (− 14.50–2.10)	− 2.90 (− 9.50–1.80)	
pH	7.32 ± 0.07	7.28 ± 0.16	n.s.
	7.34 (7.13–7.43)	7.29 (6.80–7.47)	
Hb (g/dL)	13.31 ± 2.33	11.39 ± 2.63	< 0.001
	13.60 (5.60–16.80)	11.70 (3.30–16.40)	
Hb > 12 g/dL	66.7%	33.3%	0.001
Hb ≤ 12 g/dL	37.9%	62.1%	
BE > − 5 mmol/L	57.7%	42.3%	n.s.
BE < − 5 mmol/L	66.7%	33.3%	
Brain injuries	41.7%	58.3%	n.s.
No brain injuries	56.0%	44.0%	
Associated abdominal lesions	44.8%	55.2%	n.s.
No associated abdominal lesions	60.6%	39.4%	
Trauma mechanism of injury			n.s.
-Invested pedestrian	72.7%	27.3%	
-Car	44.7%	55.3%	
-Motorbike	56.4%	43.6%	
-Bike	60.0%	40.0%	
-Precipitation	52.9%	47.1%	
-Others	50.0%	50.0%	
Contrast blush	42.4%	57.6%	0.010
No contrast blush	68.9%	31.1%	
Pseudoaneurysm	50.0%	50.0%	n.s.
No pseudoaneurysm	61.4%	38.6%	
Hemoperitoneum at TC	54.4%	45.6%	n.s.
Number of quadrants with hemoperitoneum			
- > 1	42.0%	58.0%	< 0.001
- ≤ 1	69.4%	30.6%	
INR (s)	1.12 ± 0.15	1.69 ± 1.11	0.001
	1.15 (0.66–1.38)	1.23 (1.04–5.05)	
INR > 1.5 s	23.5%	76.5%	0.014
INR < 1.5 s	55.7%	44.3%	
Fibrinogen (mg/dL)	215.52 ± 53.98	168.23 ± 67.51	0.020
	205.00 (156.0–491.00)	173.00 (26.00–260.00)	
Fibrinogen ≤ 200 mg/dL	38.1%	61.9%	0.031
Fibrinogen > 200 mg/dL	60.4%	39.6%	
PLT/mm^3^	217.38 ± 49.76	198.29 ± 83.93	n.s.
	220.00 (137.00–315.00)	190.00 (156.00–401.00)	
Positive eco-fast	33.9%	66.1%	< 0.001
Negative eco-fast	72.7%	27.3%	
RBC transfusion at the ED	34.6%	65.4%	0.032
No RBC transfusion at the ED	58.2%	41.8%	

*ISS* Injury Severity Score, *HR* heart rate, *SBP* systolic blood pressure, *ED* emergency department, *BE* base excess, *Lac* lactates, *Hb* hemoglobin, *RBC* red blood cell, *AAST* American Association for the Surgery of Trauma, *WSES* World Society of Emergency Surgery, *PLT* platelet, *INR* International Normalized Ratio

**Table 4:** Multivariate analysis of risk factors for OM at the arrival of patient at the ED

Variables	*p* value	OR
ISS > 25	n.s.	/
Contrast blush	n.s.	/
Positive e-fast	n.s.	/
RBC transfusion in ED	n.s.	/
Fibrinogen ≤ 200 mg/dL	n.s.	/
INR > 1.5 s	n.s.	/
Quadrants with hemoperitoneum > 1	n.s.	/
Hb ≤ 12 g/dL	n.s.	/
WSES IV	0.049	5.44

*ISS* Injury Severity Score, *CB* contrast blush, *ED* emergency department, *RBC* red blood cell, *SI* shock index, *AAST* American Association for the Surgery of Trauma, *Hb* hemoglobin, *WSES* World Society of Emergency Surgery, *INR* International Normalized Ratio

At the multivariate analysis, the WSES IV splenic injury grade was found as the only one risk factor for OM at the arrival of patients (OR 5.44, *p* = 0,049) (Table 4).

The risk factors for OM as a definitive treatment were analyzed, including both patients treated with OM at the arrival in the ED and patients operated for fNOM. The OM was applied on 53.2% of patients as a definitive treatment.

Risk factors emerging from univariate and multivariate analyses are shown in Tables 5 and 6.

**Table 5:** Univariate analysis for OM as a definitive treatment

Characteristics	Mean ± SD Median (range)		*p* value
	Successful NOM	OM + fNOM	
WSES I	79.5%	20.5%	< 0.001
WSES II	33.3%	66.7%	
WSES III	27.8%	72.2%	
WSES IV	13.3%	86.7%	
ASST 1	100.0%	00.0%	< 0.001
ASST 2	68.8%	31.2%	
ASST 3	35.3%	64.7%	
ASST 4	16.7%	83.3%	
ASST 5	0.0%	100.0%	
WSES I-II-III	53.2%	46.8%	< 0.001
WSES IV	13.3%	86.7%	
ASST ≤ 3	56.3%	43.7%	< 0.001
ASST > 3	14.3%	85.7%	
Age (years)	48.79 ± 17.94	51.36 ± 18.74	n.s.
	47.63 (18.00–87.00)	49.00 (17.00–91.00)	
No anticoagulant/antiplatelet drugs	39.29%	60.71%	n.s.
Anticoagulant/antiplatelet drugs	40.00%	60.00%	
HR (bpm)	85.57 ± 17.94	93.87 ± 21.32	0.039
	85.00 (48.00–133.00)	90.00 (55.00–145.00)	
HR < 120 bpm	48.00%	52.00%	n.s.
HR > 120 bpm	38.30%	61.70%	
SBP (mmHg)	122.21 ± 20.18	107.54 ± 26.57	0.001
	120.00 (70.00–170.00)	110.00 (53.00–167.00)	
SBP > 90 mmHg	50.5%	49.5%	< 0.001
SBP < 90 mmHg	13.0%	87.0%	
Shock index < 1	51.0%	49.0%	0.021
Shock index > 1	15.4%	84.6%	
ISS	21.89 ± 10.25	32.81 ± 13.02	< 0.001
	22.00 (5.00–48.00)	29.00 (9.00–75.00)	
ISS < 25	70.0%	30.0%	< 0.001
ISS > 25	26.8%	73.2%	
Lactate	2.99 ± 1.96	3.45 ± 1.78	n.s.
	2.44 (0.80–9.24)	3.08 (1.27–8.00)	
BE (mmol/L)	− 3.07 ± 3.87	− 3.40 ± 2.94	n.s.
	− 2.80 (− 14.50–2.10)	− 3.10 (− 9.50–1.80)	
pH	7.32 ± 0.08	7.29 ± 0.15	n.s.
	7.34 (7.13–7.43)	7.31 (6.80–7.47)	
Hb (g/dL)	13.78 ± 1.88	11.29 ± 2.66	< 0.001
	14.1 (10.10–16.80)	11.60 (3.30–16.40)	
Hb ≤ 12 g/dL	24.1%	75.9%	< 0.001
Hb > 12 g/dL	60.6%	39.4%	
BE > − 5 mmol/L	57.7%	42.3%	n.s.
BE < − 5 mmol/L	50.0%	50.0%	
Brain injuries	29.2%	70.8%	n.s.
No brain injuries	47.0%	53.0%	
Associated abdominal lesions	36.2%	63.8%	n.s.
No associated abdominal lesions	50.0%	50.0%	
Trauma dynamic			n.s.
-Invested pedestrian	45.5%	54.5%	
-Car	26.3%	73.7%	
-Motorbike	53.9%	46.1%	
-Bike	60.0%	40.0%	
-Precipitation	52.9%	47.1%	
-Others	42.9%	57.1%	
Contrast blush	33.3%	66.7%	0.025
No contrast blush	56.8%	43.2%	
Pseudoaneurysm	25.0%	75.0%	n.s.
No pseudoaneurysm	50.5%	49.5%	
Hemoperitoneum at CT scan	43.0%	57.0%	n.s.
Number of quadrants with hemoperitoneum at CT scan			
-> 1	30.0%	70.0%	0.001
-≤ 1	59.7%	40.3%	
INR (s)	1.11 ± 0.15	1.59 ± 1.02	0.001
	1.15 (0.66–1.38)	1.18 (1.04–5.05)	
INR > 1.5 s	11.8%	88.2%	0.002
INR < 1.5 s	48.5%	51.5%	
Fibrinogen (mg/dL)	221.06 ± 57.54	172.76 ± 62.17	n.s.
	216.00 (156.00–401.00)	175.00 (26.00–260.00)	
PLT/mm^3^	218.82 ± 47.96	200.76 ± 79.31	n.s.
	220.00 (137.00–315.00)	190.00 (55.00–302.00)	
Positive eco-fast	29.0%	71.0%	0.002
Negative eco-fast	59.1%	40.9%	
RBC transfusion at the ED	11.5%	88.5%	< 0.001
No RBC transfusion at the ED	53.0%	47.0%	

*ISS* Injury Severity Score, *HR* heart rate, *SBP* systolic blood pressure, *ED* emergency department, *BE* base excess, *Lac* lactates, *Hb* hemoglobin, *RBC* red blood cells, *AAST* American Association for the Surgery of Trauma, *WSES* World Society of Emergency Surgery, *PLT* platelet, *CB* contrast blush, *PSA* pseudoaneurysms, *INR* International Normalized Ratio

**Table 6:** Multivariate analysis of risk factors for OM as a definitive treatment

Variables	*p* value	OR
INR > 1.5 s	n.s.	/
RBC transfusion in ED	n.s.	/
Hb ≤ 12 g/dL	n.s.	/
ISS > 25	0.013	5.75
Contrast blush	n.s.	/
Positive e-fast	n.s.	/
Quadrants with hemoperitoneum > 1	n.s.	/
WSES IV	0.029	7.22

*ISS* Injury Severity Score, ED emergency department, *RBC* red blood cell, *SI* shock index, *AAST* American Association for the Surgery of Trauma, *Hb* hemoglobin, *WSES* World Society of Emergency Surgery, *CB* contrast blush, *INR* International Normalized Ratio

The WSES grade IV (OR 7.22, *p* = 0,029) and ISS value higher than 25 (OR 5.75, *p* = 0,013) were found as the only significant risk factors at the multivariate analysis (Table 6).

**Fig. 2:**
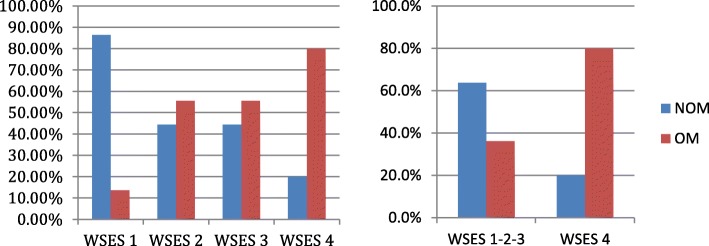
OM and NOM rate at the arrival of patient according to WSES splenic injury grade (NOM, Non Operative Management; OM, Operative Management)

**Fig. 3:**
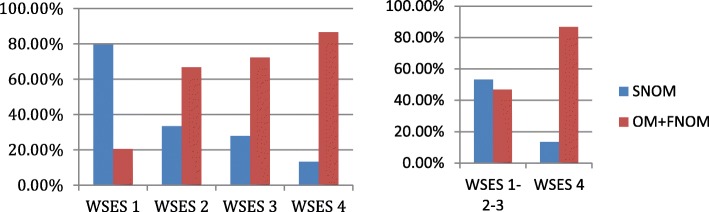
OM and NOM rate as a definitive treatment according to the WSES splenic injury grade (SNOM, Successful Non Operative Management; OM, Operative Management; FNOM, Failure of Non Operative Management)

The previous analysis showed as OM rate, both at the arrival of patient and as a definitive treatment, increased with the increasing of the WSES splenic injury grade, in particular for the WSES grade IV compared with lower grade (Figs. 2 and 3).

The present study verified also if the AAST and WSES classifications were predictive for AE at the arrival of patient with splenic injury or during hospital stay. While an AAST grade higher than 3 was not a significant risk factor for AE (AAST > 3 (20.0%) vs AAST ≤ 3 (17.2%), n.s.), a WSES splenic injury grade of III was found as a significant risk factor (WSES 3 (38.9%) vs WSES 1-2-4 (13.9%), *p* = 0.010).

## Discussion

After the introduction of AE and the modern tools in bleeding management, the NOM failure rate decreased from 23–67% to 4–42% [6–10] and it was no longer associated with the AAST injury grade (i.e., anatomical degree of lesion) [11]. So it has been accepted that the physiopathologic status of the patients, more than the anatomy of the splenic lesions, should lead the therapeutic decision in splenic trauma. Furthermore, many studies [8, 12–16] showed that the vascular lesions (CB, PSA, AVF), which have significant incidence also in low-grade injuries [12, 16], were predictive factors for NOM failure and that they should be considered indications to AE. Vascular lesions are not considered in the AAST classification. The WSES spleen trauma classification considers both the anatomical injury grade and the clinical conditions of the patients, so it can be considered as a complete tool to lead splenic trauma management, especially if associated to dedicated guidelines. From the analysis emerged, all the factors related to OM and fNOM are those linked to the physiology of the patients and more than the anatomy. AAST classes related to the OM + fNOM mainly for the anatomical basis that represents a proxy even of the physiological conditions. WSES classes consider even the physiology from the beginning, and in fact, the patient stratification is slightly different (Table 5).

Actually, in fact, the possibility to not operate spleen trauma and to manage them with NOM is becoming mandatory in right patients and in all those systems where enough facilities are present. The NOM percentage can furthermore be considered as a proxy of the preparedness of the system to manage with severe trauma with advanced strategies, allowing preserving as many patients as possible from operative procedures. To obtain this result is necessary to set a system where classification and management of traumatized patients are driven by updated patient stratification tool and guidelines. Present classification associated to the last released guidelines might definitively allow for an improvement in spleen injured patient management. As showed in the analysis, in fact, it more strictly adheres to the necessities of the common clinical practice. As a counterpart, however, the variability within the different members even from a single department accounts for the real life data.

Population of the present study represents the typical case mix of two Italian trauma centers. The cases presented in Italy are the most part victim of blunt trauma. In general, few penetrating traumas are treated in Italian hospitals. The NOM rate reported in literature ranged from 60 to 95% [17–20] and includes both studies conducted in structures with local protocols for splenic trauma management and study conducted in structures in which trauma management was based on the single surgeon experience and common sense. Present study renders the actual situation in management of splenic injury in trauma centers without the application of a shared guideline, and so it gives a good representation of the real situation. The NOM rate is 53.2%, and it can be considered a not-high rate. In fact, even patients with low injury grade were splenectomized. Present data showed, even in this context, as the WSES spleen injury grade IV is a significant risk factor for OM, both at the arrival of the patients and as a definitive treatment. Furthermore, a WSES spleen injury grade III is a risk factor for AE (WSES 3 (38.9%) vs WSES 1-2-4 (13.9%), *p* = 0.010). WSES grade IV represents the only factor related to the OM as management at the patient admission. In fact, the hemodynamic status is the only determinant of the necessity to proceed to operating room. The anatomical grade of damage is not influent on the emergency management in presence of hemodynamic instability at admission. However, the relative high OM rate, also in lower injury grade (OM rate is 36.2% in WSES I,I, and III injury grade), reflects the need for standardized and widely shared guideline in order to increase conservative management. Even if in presence of such a big variability in patient management, the WSES classification showed to be effective in driving the management. Therefore, the benefits deriving from the use the WSES trauma spleen classification could have their greatest expression if associated with the application of the widely approved WSES spleen trauma guidelines. Their combined large-scale application could realistically increase successful NOM rate and improve the spleen trauma management.

The limitations of this study are that this is an observational study, even if prospective, and that patients did not have isolated spleen injury and so the associated lesions could have partially influenced results; however, as said, it reports the reality in the trauma centers’ daily practice. As a counterpart, however, this study stresses the necessity to diffuse and apply a common way to proceed. This will allow to reduce the number of operated patients and to improve the management quality by reducing even the short- and long-term morbi-mortality of unnecessary laparotomies and splenectomies.

## Conclusions

The WSES classification is a good and reliable tool in the decision-making process in splenic trauma management.

## Data Availability

Not applicable

## Abbreviations

AAST: American Association for the Surgery of Trauma
AE: Angioembolization
AG: Angiography
ASA: American Society of Anesthesiologists
AVF: Arterovenous fistula
BE: Base excess
CB: Contrast blush
ED: Emergency department
fNOM: Failure of non-operative management
HR: Heart rate
INR: International normalized ration
LAC: Lactates
NOM: Non-operative management
OIS: Organ Injury Severity Score
OM: Operative management
OR: Odds ratio
PSA: Pseudoaneurysm
RBC: Red blood cell
RR: Risk ratio
SBP: Systolic blood pressure
SI: Shock index
SNOM: Successful non-operative management
TC: Trauma center
WSES: World Society of Emergency Surgery
